# Collaborative Inference for Accelerated Failure Time Model Using Clinical Center‐Level Summary Statistics

**DOI:** 10.1002/sim.70279

**Published:** 2025-10-22

**Authors:** Mengtong Hu, Xu Shi, Ziyang Gong, Peter X.‐K. Song

**Affiliations:** ^1^ Department of Biostatistics University of Michigan Ann Arbor Michigan USA; ^2^ Center for Statistical Research Southwestern University of Finance and Economics Chengdu China

**Keywords:** data privacy, distributed inference, meta‐analysis, renewable estimation, survival analysis

## Abstract

Multi‐center clinical research offers numerous advantages, including the ability to obtain a larger combined sample size and to reduce center‐specific insufficiency and imbalance in event rates, ultimately leading to more robust and generalizable findings. This paper develops a collaborative analytic framework for survival data analysis using summary statistics and the Accelerated Failure Time (AFT) model, a popular alternative to the Cox proportional hazards model for the analysis of time‐to‐event data. The AFT model directly accounts for the effects of the covariates on times to event, rather than through hazard functions, with no proportionality assumption required compared to the Cox model. Given that it bypasses the construction of partial likelihood, it gives rise to more flexibility in integrative analyses of survival data collected from multiple clinical sites. Our proposed distributed inference method focuses on a class of parametric AFT models with Weibull, log‐normal, and log‐logistic distributions for time‐to‐event outcomes, with a distributed likelihood ratio test established under the generalized gamma distribution to assess the goodness‐of‐fit across different candidate parametric AFT models. We present large‐sample properties for the proposed distributed method and illustrate its performance through simulation experiments and a real‐world data example on kidney transplantation.

## 1 Introduction

There have been growing appeals for developing privacy‐aware survival analysis methods for multi‐center clinical studies without the need to merge subject‐level data into a centralized database. In such a way, center‐specific raw data are not shared with outside researchers to control the scope and boundaries of data sharing. In this paper, the outcome of interest is the time until an event or failure occurs, and censoring on the outcome is commonly encountered when a subject drops out of a study before the observation period ends. Special considerations are needed to extend existing distributed learning methods to conduct estimation and inference for the effects of covariates on survival outcomes. Some recent efforts have been centered around the distributed implementation of partial likelihood estimation for the Cox Proportional Hazards (PH) model, assuming that subject‐level time‐to‐event information and aggregated information on the risk‐set level can be directly shared across study centers [1, 2, 3, 4]. There have been known risks of information leakage when sharing aggregated at this granularity, and thus it is not satisfactory given real‐world patient privacy concerns [5]. The Accelerated Failure Time (AFT) model, popular in industrial settings for modeling time‐to‐failure data, presents a desirable alternative [6, 7].

Although not as widely used in medical research as the Cox PH model, the AFT model directly accounts for the effects of the covariates on times to events, rather than on hazard functions, making it easier to interpret without assuming proportional hazards. The classical Cox model assumes that the hazard ratio between any two individuals is constant throughout, although the hazard itself may change over time, whereas the AFT model does not rely on the PH assumption and allows for time‐varying covariate effects. In addition to assuming that the covariates have a direct multiplicative effect on the hazard function, the AFT model assumes that the covariates also modify the time scale. As shown in the literature, the AFT model may provide a better fit for many clinical survival data than the Cox PH model [8, 9]. In this paper, we aim to develop privacy‐aware distributed collaborative estimation and inference for AFT models. We avoid the sharing of raw risk‐set level time‐to‐event data across centers as part of the partial likelihood estimation, and instead, our method is based on full likelihood, which only requires the sharing of center‐level summary statistics.

The parameters in an AFT model can be estimated using various methods, such as parametric estimation and rank‐based methods [7, 10, 11]. To our knowledge, no previous research has explored the federated estimation of the AFT model in the context of multicenter clinical studies when a centralized analysis is not possible due to the prohibition of pooling raw data from centers into a centralized database. Recently, a related work by Chen et al. [12] has proposed an efficient distributed rank‐based algorithm within a divide‐and‐conquer framework for the estimation and inference of the AFT model for massive datasets. Their approach allows for efficient AFT estimation by dividing the data into smaller subsets stored on a local machine so that a meta‐type estimation can be carried out. Albeit being distributed, this method has two major limitations when applied to perform a distributed inference. First, the divide‐and‐conquer results rely on reliable local AFT estimation, which is likely to be of poor quality given varying recruitment capacity across centers; at some study sites, small sample sizes or low sample variability can reduce the overall statistical power of the meta‐analysis under the divide‐and‐conquer framework. Moreover, their method requires iterative updates and multiple rounds of cross‐center communications, leading to more computational costs and implementation difficulty in practice. An alternative approach based on the classical inverse variance‐weighted site‐specific AFT parameter estimates is a solution with no need for iterations for estimating the global AFT model coefficients (e.g., Cochran [13]). This fixed‐effects meta‐analysis (Meta‐FE) approach saves a substantial amount of computing resources, which is termed the classical meta‐analysis in this paper. The random‐effects meta‐analysis (Meta‐RE) approach serves as an alternative to accommodate heterogeneity across sites through random effects, which is known to appear conservative when modest or less heterogeneity occurs among the true site‐specific effects [14]. It is worth pointing out that meta‐analysis only retains the optimal statistical power when the sample size from each local site is sufficiently large [15]. There have been several distributed inference methods that aimed to improve the meta‐analysis methodology, such as the communication‐efficient method using the local Hessian estimators proposed by Jordan et al. [16], which, however, requires comparable local sample sizes (i.e., all local sample sizes grow at the same rate as the total combined sample size) to attain the optimal convergence rate at the order of the total sample size. Yin [17] improved the convergence rate by combining the M‐estimator and the local gradients, albeit still depending on local sizes. Chen et al. [18] proposed an iterative first‐order Newton‐type estimator to further improve the convergence rate, which unfortunately requires multiple rounds of communication between centers. Compared to these approaches, our proposed method achieves desirable statistical efficiency with a convergence rate at the order of the total sample size, with no need for iterations and no specific requirements on the local sample size.

Our proposed method, *Collaborative Accelerated Failure Time Analysis* (cafta), is designed to overcome these drawbacks by extending a serial updating framework recently developed in Hu et al. [5, 19] The collaborative inference framework is inspired by renewable estimation, proposed by Luo and Song [20] for the analysis of independent streaming data batches in generalized linear models. Luo et al. [21] later extended this approach to accommodate batch‐varying effects and time‐varying effects in streaming longitudinal data settings [22]. Wang et al. [23] develop an online learning approach for time‐varying effect estimation while screening for abnormal data batches. In contrast, our cafta focuses on time‐to‐event analysis under the distributed storage of independent datasets at different clinical centers, assuming no data‐sharing between sites. To avoid ranking residuals across sites due to semi‐parametric AFT estimation, in this paper, we consider the parametric AFT estimation that only requires the first site in the sequence to be sufficiently large. Our proposed method enables a class of parametric AFT models with Weibull, log‐normal, and log‐logistic distributions, for which a collaborative likelihood ratio test is established under the generalized gamma distribution to assess the goodness‐of‐fit across different AFT models.

To illustrate the utility of the proposed cafta methodology, we explore the biological and socioeconomic factors associated with 5‐year death‐censored graft failures in patients who received kidney transplants across the United States. We leverage the data from the Scientific Registry of Transplant Recipients (SRTR), managed by the Organ Procurement and Transplantation Network (OPTN). The OPTN is organized into 11 geographic regions that oversee transplant operations across the U.S. Our analysis considers a scenario in which patient data from individual regions cannot be pooled or centralized. cafta enables us to draw conclusions about the entire U.S. population represented by all regions in OPTN without the need for sharing of individual‐level data.

The organization of this paper is as follows. Based on the summary of AFT models in Section 2, we introduce the distributed inference methodology and goodness‐of‐fit test in Section 3. Section 4 establishes the asymptotic properties of our proposed method. Section 5 presents the simulation results of our method. Section 6 illustrates the proposed method on real‐world data from the Scientific Registry of Transplant Recipients (SRTR). Section 7 provides a discussion on the method. Some technical details are included in the Supporting Information.

## 2 Parametric Accelerated Failure Time Models

### 2.1 Parametric AFT Models

We follow the notations for the AFT model in Kalbfleisch and Prentice [7]. Let (T,X) be the set of failure time (denoted by T) and the p‐element vector of covariates (denoted by X). The AFT model specifies the logarithm of the failure time, log(T), as a linear function of the covariates X given as follows:

(1) $$\begin{array}{l} log(T)=\alpha +X^{⊤}\beta +\sigma W, \end{array}$$

where the intercept α is the location parameter, β is the association parameters of interest, and σ is the scale parameter. The error variable W follows a parametric distribution with density f(w). Various distributions of W correspond to different AFT models. A Weibull AFT model refers to the case of $f(w)=e^{\left(w-e^{w}\right)}$, a log‐logistic model is given by $f(w)=e^{w}{\left(1+e^{w}\right)}^{-2}$, and the log‐normal regression model is $f(w)={\left(2\pi \right)}^{-1/2}e^{-w^{2}/2}$. These three types of AFT models are considered in this paper.

Alternatively, the AFT model may be expressed by a survival function, which is commonly seen in the Cox PH model parameterization. Let $S_{0}^{*}(w)$ denote the survival function of exp(σW). Then the survival function of T under the model in Equation (1) is

$$\begin{array}{l} S(t;X)=S_{0}^{*}\left[\exp(-x^{⊤}\beta )t\right],\;t\geq 0. \end{array}$$

Clearly, compared to the Cox PH model that assumes multiplicative covariate effects on the baseline hazard, the AFT model characterizes the influence of the covariates accelerating (when $x^{⊤}\beta <0$) or decelerating (when $x^{⊤}\beta >0$) the failure time multiplicatively. Of note, under the Weibull AFT model, the regression coefficients for the AFT and Cox PH model are related by a factor of −σ, but such a relation does not hold for log‐logistic or log‐normal AFT models.

### 2.2 AFT Modeling Under Generalized Gamma Distribution

The error term densities f(w) for Weibull and log‐normal AFT models may be unified under the following generalized gamma (GG) distribution [24]. In fact, as we detail in Section 3.3, the GG distribution is useful for the discrimination of competing parametric models [24, 25]. With one additional parameter q, the distribution of the error term W is

(2) $$\begin{array}{l} f(w;q)=\left\{\begin{array}{ll} \left|q|{\left(q^{-2}\right)}^{q^{-2}}\exp[q^{-2}\{qw-\exp(qw)\}]/\Gamma (q^{-2}\right) & ,q\neq 0\\ {\left(2\pi \right)}^{-1/2}\exp(-\frac{1}{2}w^{2}) & ,\;q=0 \end{array}\right. \end{array}$$

where Γ(z) denotes the gamma integral, $\Gamma (z)=\int_{0}^{\infty }t^{z-1}e^{-t}\;dt$. When q=0, the density in Equation (2) is the normal distribution, which corresponds to the log‐normal AFT model; when q=1, the density in Equation (2) gives the Weibull AFT model. Thus, the Weibull and log‐normal models are two special cases within the family of GG AFT models. A certain goodness‐of‐fit assessment may be used via the value of q to determine a more suitable parametric AFT model for the data through, for example, the likelihood ratio test or Wald test on candidate q values.

## 3 Multicenter AFT Models for Distributed Data

### 3.1 Centralized Analysis

Consider a random sample of N individuals independently sampled from K clinical sites, indexed by k=1,…,K, each site having a sample size of $n_{k}$. For the ith individual from site k, we observe a vector of baseline covariates $X_{ki}$, and an observed time $Y_{ki}:=min(T_{ki},C_{ki})>0$, where $T_{ki}$ and $C_{ki}$ are the event time and censoring time, respectively. Let $\Delta_{ki}:=I(T_{ki}\leq C_{ki})$ be the event indicator. We denote the kth site‐specific data as ${𝒪}_{k}=\{{𝒪}_{ki}=(X_{ki},Y_{ki},\Delta_{ki}):i\in \{1,\ldots ,n_{k}\}\}$. We assume that triplets ${𝒪}_{ki}$ for $i\in \{1,\ldots ,n_{k}\}$ and k∈{1,…,K} are independent and identically distributed (i.i.d.) observations drawn from the same underlying population, and the censoring is noninformative. Let $W_{ki}=\left\{\log(T_{ki})-\alpha -X_{ki}^{⊤}\beta \right\}/\sigma$. We focus on the distribution f(·) of $W_{ki}$ to be either a distribution discussed in Section 2.1 or GG. Let θ=(α,β,σ) be the model parameters for Weibull, log‐logistic, and log‐normal AFT models, and with slight abuse of notation, let θ=(α,β,σ,q) for the GG model. Ideally, if individual‐level data from all sites could be merged into a centralized database, the overall logarithm of the likelihood across all K sites would be given by

(3) $$\begin{array}{ll} \ell (\theta ) & =\overset{K}{\underset{k=1}{\sum }}\overset{n_{k}}{\underset{i=1}{\sum }}\ell_{ki}(\theta )=\overset{K}{\underset{k=1}{\sum }}\overset{n_{k}}{\underset{i=1}{\sum }}\left\{\Delta_{ki}\log f(w_{ki})-\log\sigma \right\}\\ & \;+(1-\Delta_{ki})\log S(w_{ki}) \end{array}$$

where $S(w_{ki})=\int_{w_{ki}}^{\infty }f(u)du$. The score function takes the form:

$$U(\theta )=\overset{K}{\underset{k=1}{\sum }}U_{k}(\theta )=\overset{K}{\underset{k=1}{\sum }}\overset{n_{k}}{\underset{i=1}{\sum }}U_{ki}(\theta )=\overset{K}{\underset{k=1}{\sum }}\overset{n_{k}}{\underset{i=1}{\sum }}\frac{\partial \ell_{ki}(\theta )}{\partial \theta }.$$

The analytical form of the score function for the Weibull AFT model, the log‐logistic AFT model, or the log‐normal AFT model can be found in Chapter 3.6 in Kalbfleisch and Prentice [7] as well as in Supporting Information: Appendix A. If the centralized data of sample size $N_{K}$ is available with permission to combine site‐specific data from $O_{1}$ to $O_{K}$ of sample size $N_{K}$ and $N_{K}=\sum_{j=1}^{j=K}n_{j}$, the oracle estimate for the parameter θ can be found as the root of U(θ)=0, denoted by ${\hat{\theta }}_{N_{k}}^{ora}$, using a certain numerical search algorithm. Due to concerns about patient privacy and strict privacy regulations, sharing individual‐level data among different sites is often unfeasible. Consequently, centralized analysis can not be easily conducted. Of note, the i.i.d. assumption is imposed for the centralized sampling protocol of the whole data, although “identically distributed” may be violated across local sites when the whole data is stored in a distributed fashion. In other words, one could hypothetically pool local datasets together and analyze the pooled data in a centralized operation under the i.i.d. assumption, despite sampling discrepancies at local sites. In the simulation study, we demonstrate the robustness and stability of the proposed method with cross‐site sampling biases, including center‐specific shifts in event rates and covariate distributions.

### 3.2 Incremental Estimation

To address the data‐sharing challenge, we propose an estimation method called *Collaborative Accelerated Failure Time Analysis* (cafta) that enables an incremental estimation in an AFT model when pooling the entire raw data from multiple sites is prohibited in a practical setting. This approach does not require sharing any individual‐level raw data across all participating sites but only certain site‐specific summary statistics. Specifically, given a set of K study sites, an incremental estimator obtained at site k, denoted by ${\hat{\theta }}_{k}$, is sequentially updated over the first k sites. The estimates of the sensitivity matrix and variability matrix are also updated sequentially by cafta. We denote the sensitivity and variability matrix, respectively, evaluated at a local site j∈{1,…,K}, respectively, as follows:

$$H_{j}({\hat{\theta }}_{j})=\overset{n_{j}}{\underset{i=1}{\sum }}-{\left.\frac{\partial U_{ji}(\theta )}{\partial \theta^{⊤}}\right|}_{\theta ={\hat{\theta }}_{j}},\;V_{j}({\hat{\theta }}_{j})=\overset{n_{j}}{\underset{i=1}{\sum }}{\left.U_{ji}(\theta )U_{ji}^{⊤}(\theta )\right|}_{\theta ={\hat{\theta }}_{j}}.$$

Obviously, ${\hat{\theta }}_{1}$ is the same as a local estimator as a root of the score equation $\sum_{i=1}^{n_{1}}U_{1i}(\theta )=0$ with the local data of ${𝒪}_{1}$. After obtaining summary statistics $\{{\hat{\theta }}_{1}$, $H_{1}({\hat{\theta }}_{1})$, $V_{1}({\hat{\theta }}_{1})\}$ from site 1, cafta passes these summary statistics to site 2. We then update ${\hat{\theta }}_{1}$ to ${\hat{\theta }}_{2}$ by solving the following renewable estimating equation proposed by Luo and Song [20] using data of site 2, that is, ${𝒪}_{2}$:

$$\overset{n_{2}}{\underset{i=1}{\sum }}U_{2i}({\hat{\theta }}_{2})+H_{1}({\hat{\theta }}_{1})({\hat{\theta }}_{1}-{\hat{\theta }}_{2})=0,$$

or equivalently, maximizing the following penalized likelihood function with respect to $\theta_{2}$: ${\hat{\theta }}_{2}=\arg\;\max_{\theta_{2}}\left\{\sum_{i=1}^{n_{2}}\ell (\theta_{2};{𝒪}_{2i})+\frac{1}{2}{\left({\hat{\theta }}_{1}-\theta_{2}\right)}^{⊤}H_{1}({\hat{\theta }}_{1})({\hat{\theta }}_{1}-\theta_{2})\right\}.$ Repeating this renewable updating scheme, cafta runs sequentially over all sites to produce both sequences of estimates and inferential quantities. In particular, ${\hat{\theta }}_{k-1}$ is updated to ${\hat{\theta }}_{k}$ at site k with ${\hat{\theta }}_{k}$ satisfying the following estimating equation:

(4) $$\overset{n_{k}}{\underset{i=1}{\sum }}U_{ki}({\hat{\theta }}_{k})+\overset{k-1}{\underset{j=1}{\sum }}H_{j}({\hat{\theta }}_{j})({\hat{\theta }}_{k-1}-{\hat{\theta }}_{k})=0$$

The estimating function in Equation (4) consists of two parts: The first term $\sum_{i=1}^{n_{k}}U_{ki}({\hat{\theta }}_{k})$ is the score function based on the local data at site k, while the second term assembles the cumulative summary statistics preceding from all previous k−1 sites. The Newton‐Raphson algorithm is applied to numerically find a solution ${\hat{\theta }}_{k}$. Given that the likelihood function for the GG model is complicated with the distribution function (2) plugged into the likelihood function (3), numerical approximations are used for the GG score function and its gradient.

For statistical inference, along with the updating steps, we also sequentially compute the cumulative sensitivity and variability matrices over the first k sites evaluated at a given point estimate ${\hat{\theta }}_{k}$. For example, if we update the sensitivity and variability matrices along with the incremental estimates ${\hat{\theta }}_{k}$, then at site k, the sensitivity matrix is given by $\sum_{j=1}^{k}H_{j}({\hat{\theta }}_{j})$ and the variability matrix is given by $\sum_{j=1}^{k}V_{j}({\hat{\theta }}_{j}).$ At the end of the updating procedure, we compute the estimated covariance matrix as ${\left\{\sum_{j=1}^{k}H_{j}({\hat{\theta }}_{j})\right\}}^{-1}$ or the robust version using the sandwich formula as ${\left\{\sum_{j=1}^{k}H_{j}({\hat{\theta }}_{j})\right\}}^{-1}\{\sum_{j=1}^{k}V_{j}({\hat{\theta }}_{j})\}{\left\{{\left[\sum_{j=1}^{k}H_{j}({\hat{\theta }}_{j})\right]}^{-1}\right\}}^{⊤}.$

Notice that the above procedures are applicable to the class of AFT models introduced in Sections 2.1 and 2.2. The details of the specific likelihood functions and score functions, as well as sensitivity and variability matrices, are given in Supporting Information: Appendix A. In practice, we may assess candidate AFT models such as those four models mentioned above, followed by a model diagnosis procedure (e.g., likelihood ratio test) described in Section 3.3 to select the most appropriate model.

### 3.3 Likelihood Ratio Test via Summary Statistics

We establish a likelihood ratio test to make a choice between two candidates, the Weibull AFT and log‐normal AFT models as special cases of the GG AFT model. We use the subscripts to denote different estimates $\hat{\theta }$ under different error distributions. For the Weibull case, the likelihood ratio test statistic is $\Lambda_{\text{Weibull}}=-2\{\ell ({\hat{\theta }}_{\text{Weibull}})-\ell ({\hat{\theta }}_{\text{GG}})\}$, where the null hypothesis is $H_{0}:q=1$ (Weibull model). For the log‐normal case, the likelihood ratio test statistics is $\Lambda_{\text{log‐normal}}=-2\{\ell (\theta_{\text{log‐normal}})-\ell (\theta_{\text{GG}})\}$, where the null hypothesis is $H_{0}:q=0$ (log‐normal model). The test statistic follows $\chi^{2}$ distribution with 1 degree of freedom asymptotically under the null [25]. Both test statistics can be computed by means of the plug‐in method via estimate ${\hat{\theta }}_{k}$, resulting in $\Lambda_{k,\text{Weibull}}$ and $\Lambda_{k,\text{log‐normal}}$. Moreover, if $\Lambda_{k,\text{Weibull}}$ (or $\Lambda_{k,\text{log‐normal}}$) exceeds the critical value from the $\chi^{2}$ distribution with 1 degree of freedom (3.84 at type I error α=0.05), we reject the null hypothesis of $H_{0}:q=1$ (or $H_{0}:q=0$). For models that are not nested in the GG family, such as the log‐logistic AFT model, the Akaike information criterion (AIC) can be used to distinguish between different parametric models.

## 4 Theoretical Guarantees

Recall that $N_{k}$ is the cumulative sample size for the first k sites, that is, $N_{k}=\sum_{j=1}^{k}n_{j}$. We discuss the large sample proprieties of our cafta estimators as $N_{k}\to \infty$, instead of $\min_{j\in \{1,\ldots ,k\}}n_{j}\to \infty$ in the parallel computing paradigm. Clearly $N_{k}\to \infty$ if $n_{j}\to \infty$ at one of the sites, or when the number of sites k→∞. We focus on the former scenario as it aligns with the multicenter study investigated in this paper. Denote the $L_{2}$‐norm of a vector u by ‖u‖. Let ${ℬ}_{\rho }(\theta_{0})=\{\theta :\|\theta -\theta_{0}\|\leq \rho \}$, ρ>0 be a compact neighborhood of size ρ around the true value $\theta_{0}$. We assume the following regularity conditions for the score function $U_{k}(\theta )$ for each site k to establish some key asymptotic properties.

Assumption 1 (Regularity Conditions) The following regularity conditions for each site k hold.

a. The score function $U_{k}(\theta )$ is continuously differentiable for all θ in the neighborhood ${ℬ}_{\rho }(\theta_{0})$;
b. the sensitivity matrix $H_{k}(\theta )$ is positive definite for all $\theta \in {ℬ}_{\rho }(\theta_{0})$;
c. the sensitivity matrix $H_{k}(\theta )$ is Lipschitz continuous for all θ∈ ${ℬ}_{\rho }(\theta_{0})$.

Conditions 1a–c are mild regularity conditions needed for legitimate asymptotic behaviors of the cafta estimator ${\hat{\theta }}_{k}$ under the classical theory of maximum likelihood estimation and their score functions.

Theorem 1 *Under the regularity conditions* *1a–c*, *the* cafta *estimator* ${\hat{\theta }}_{k}$ *is consistent for the true value* $\theta_{0}$, *that is*, ${\hat{\theta }}_{k}\overset{p}{\to }\theta_{0}$, *as* $N_{k}\to \infty$.

Theorem 2 *Under the regularity conditions* *1a–c*, *the* cafta *estimator* ${\hat{\theta }}_{k}$ *is asymptotically normally distributed, that is*, $\sqrt{N_{k}}({\hat{\theta }}_{k}-\theta_{0})\overset{d}{\to }𝒩(0,J(\theta_{0})),\;\;\;\text{as}\;N_{k}\to \infty$, *where* $J(\theta_{0})={\left\{\sum_{j=1}^{k}H_{j}(\theta_{0})\right\}}^{-1}\{\sum_{j=1}^{k}V_{j}(\theta_{0})\}{\left\{{\left[\sum_{j=1}^{k}H_{j}(\theta_{0})\right]}^{-1}\right\}}^{⊤}$ *is the robust sandwich covariance matrix*.

It follows from Theorem 2 that the proposed cafta estimator takes advantage of the combined sample size; that is, the convergence rate of the cafta estimator ${\hat{\theta }}_{k}$ is $O_{p}(N_{k}^{-1/2})$, whereas the convergence rate of the local estimator at the kth site is $O_{p}(n_{k}^{-1/2})$. Note that the local convergence rate $O_{p}(n_{k}^{-1/2})$ is not equivalent to the cumulative convergence rate $O_{p}(N_{k}^{-1/2})$ unless $n_{k}/N_{k}=O(1)$ for all k.

Theorem 3 *Under the regularity conditions* *1a–c*, *the* cafta *estimator* ${\hat{\theta }}_{k}$ *and the oracle estimator* ${\hat{\theta }}_{N_{k}}^{ora}$ *are asymptotically equivalent, in the sense that* $\left\|{\hat{\theta }}_{k}-{\hat{\theta }}_{N_{k}}^{ora}{\|}^{2}=o_{p}(N_{k}^{-1}\right)$ *as* $N_{k}\to \infty$.

Theorem 3 implies that the asymptotic difference between ${\hat{\theta }}_{k}$ and ${\hat{\theta }}_{N_{k}}^{ora}$ is $o_{p}({N_{k}}^{-1/2})$, and thus they are stochastically equivalent in the sense that they have the same asymptotic normal distribution. This implies that the cafta estimator is as efficient as the oracle estimator.

Theorem 4 *Under the regularity conditions* *1a–c* *and the null hypothesis that* $H_{0}:q=q_{0}$, *the likelihood ratio test statistic* $\Lambda_{k,\text{GG}(q_{0})}$ *follows asymptotically a* $\chi^{2}$ *distribution with* 1 *degree of freedom as* $N_{k}\to \infty$ *. Consequently, under the null when* $q_{0}=1$ (*Weibull*) *or* $q_{0}=0$ (*log‐normal*), *the* $\Lambda_{k,\text{Weibull}}\overset{d}{\to }\chi_{1}^{2}$ *or* $\Lambda_{k,\text{Log‐normal}}\overset{d}{\to }\chi_{1}^{2}$ *as* $N_{k}\to \infty$.

Theorem 4 establishes the validity of the likelihood ratio test and justifies the rejection rules under the $\chi^{2}$ distribution. The proofs for the above theorems can be found in Supporting Information: Appendix B.

## 5 Simulation Experiments

### 5.1 General Setup

We evaluate the finite‐sample performance of the proposed cafta method for a class of parametric AFT distributions. We first generate the full data under a certain assumed distribution for time to event, then randomly split the data into subsets, one for each study site. We consider K (K=6) number of sites with fixed sample sizes of 500,300,100,50,50,50, which mimics the common real‐life scenarios where some participating sites have small sample sizes. To mimic the covariate distributions in the SRTR data example discussed later in Section 6, we generate four covariates $X_{1}$ to $X_{4}$ with some correlations: $\left(X_{1},X_{2})∼{𝒩}_{2}(\text{0},\sum_{0}\right)$ where the variances in $\sum_{0}$ are 1 and the covariances are 0.3; $X_{3}∼\text{Bernoulli}(0.8)$ and $X_{4}|X_{3}=0∼\text{Multinomial}(0.2,0.2,0.3,0.3)$ or $X_{4}|X_{3}=1∼\text{Multinomial}(0.1,0.2,0.4,0.5)$. For convenience, we use $X={\left(X_{1},X_{2},X_{3},X_{4}\right)}^{⊤}$ where $X_{4}$ itself is a three‐dimensional vector that represents four categories through three dummy variables. The true values of the β coefficients are set at (0.15,−0.15,0.3,0.1,−0.1,0.3) where the third to sixth values are the coefficients for the dummy variables corresponding to the second, third, and fourth categories with respect to the reference (i.e., first) category of $X_{4}$. We set the scale parameter, σ, and intercept parameter α in the AFT model (1) at 0.8 and 0.3, respectively. The logarithm of the event time T is generated through the model specification in the AFT model (1) with a certain error distribution as described in Section 2.1. The censoring time C is independently generated from a uniform distribution Unif(0,ζ) with a varying right limit ζ empirically determined such that the event rate is controlled to be around 10%, 30%, or 50%.

For each of the three error distributions below, we compare the estimation results of the proposed cafta method to those of the oracle estimation method, which performs a centralized analysis, and to the inverse variance weighted meta‐analysis estimation method—also known as fixed‐effect meta‐analysis (denoted as Meta‐FE)—implemented within the divide‐and‐conquer parallelized paradigm. Due to space limitations, we choose to present results for $\beta_{1}$, the coefficient for the continuous variable $X_{1}$, and $\beta_{6}$, the coefficient for the fourth category of $X_{4}$. The results for the remaining β coefficients exhibit similar performance and are included in Supporting Information: Appendix C.1.

To further assess the robustness of our method against center‐specific heterogeneity due to certain distributional shifts across sites, we conducted additional simulations involving varying event rates across sites and center‐specific covariate distribution shifts. The results of these simulation experiments are reported in Supporting Information: Appendices C.2 and C.3, along with the results from both the fixed‐effects meta‐analysis (Meta‐FE) and the random‐effects meta‐analysis (Meta‐RE). The latter is routinely used in the meta‐analysis when there exists strong center‐specific heterogeneity among local covariate effects. In summary, CAFTA maintains a reasonable coverage close to the nominal level and comparable to that of the oracle method across all cases of error distributions considered. In comparison, the Meta‐FE method exhibits increased bias and undercoverage, while the Meta‐RE method yields enlarged variance estimates and overcoverage, implying a conservative type I error control and thus power loss.

#### 5.1.1 Results From Weibull AFT Model

For the simulation experiment under the Weibull AFT model, the errors are generated through a standard extreme value distribution. As shown in Table 1, the oracle and cafta have almost identical performances when the event rate is 30% or 50%. When the event rate is as low as 10%, the oracle method has slightly worse numerical performance than cafta demonstrated by higher MSE and a higher failure convergence rate (higher number of experiments yielding non‐converging results). This is likely due to poor starting values used by the *R* package *survival* in some repetitions, causing the likelihood estimation to converge into local minima, especially when the event rate is low. In contrast, the cafta method adds variability during updates to the gradient calculation, which facilitates the optimization process to avoid getting trapped in local minima. This additional variability comes from the data being collected at different sites, which is similar to the trick of mini‐batch stochastic gradient descent, where the gradients used in updates are calculated separately on each batch of data obtained by a random split of the training data. The meta‐analysis method appears less reliable, especially since its coverage probability (CP) appears to be much lower than the nominal level. It has the largest mean squared bias relative to the true β (MSE) and greater relative bias to the oracle estimate (ARB) than cafta in all scenarios. The differences between MSE and the empirical standard error (ESE) in the meta‐estimation indicate that its variance calculation is of poor quality. Moreover, the type I error for the likelihood ratio test $\Lambda_{\text{Weibull}}$ for $H_{0}:q=1$ is well controlled at 6.3%, 4.9%, and 4.8% for event rates of 10%, 30%, and 50%, respectively.

**TABLE 1:** Simulation results from the Weibull AFT model summarized by all cases of successful convergence over 3000 repetitions. K=6 with the fixed updating order of $n_{1}=500$, $n_{2}=300$, $n_{3}=100$, $n_{4}=50$, and $n_{5}=50$. The true values of $\beta_{1}=0.15$ and $\beta_{6}=0.3$. Refer to the footnotes for the definitions of each metric.

		$\beta_{1}(0.15)$			$\beta_{6}(0.3)$		
Event rate	Metrics	Oracle	CAFTA	Meta	Oracle	CAFTA	Meta
10%	ARB(%)	0.0	10.7	94.5	0.0	29.0	243.6
	CP(%)	96.0	95.6	91.6	95.4	95.2	90.4
	$\text{MSE}\times 10^{-3}$	6.53	6.25	16.8	78.0	70.5	491
	$\text{ASE}\times 10^{-2}$	8.25	8.06	8.01	27.2	26.5	27.5
	$\text{ESE}\times 10^{-2}$	8.08	7.88	41.0	27.9	26.5	222
	fails(#)	47	30	2214	47	30	2214
30%	ARB(%)	0.0	4.1	19.3	0.0	8.2	52.6
	CP(%)	94.5	94.5	93.7	94.9	94.8	92.5
	$\text{MSE}\times 10^{-3}$	2.34	2.32	2.56	25.5	24.8	29.2
	$\text{ASE}\times 10^{-2}$	4.79	4.76	4.75	15.6	15.5	15.3
	$\text{ESE}\times 10^{-2}$	4.84	4.81	5.05	16.0	15.7	17.0
	fails(#)	0	0	176	0	0	176
50%	ARB(%)	0.0	0.9	7.0	0.0	3.0	31.6
	CP(%)	94.9	94.9	93.1	94.2	94.0	90.8
	$\text{MSE}\times 10^{-3}$	1.37	1.37	1.50	15.2	15.1	18.8
	$\text{ASE}\times 10^{-2}$	3.69	3.67	3.65	12.0	11.9	11.6
	$\text{ESE}\times 10^{-2}$	3.70	3.70	3.87	12.3	12.3	13.5
	fails(#)	0	0	51	0	0	51

*Note:* ARB average absolute relative bias with respect to the oracle estimate; CP, coverage probability; MSE, mean squared bias; ASE, mean estimated standard error of the estimates; ESE, empirical standard error; fails, number of repetitions when oracle and cafta failed to converge and when at least one site failed to converge for meta.

#### 5.1.2 Results From the Log‐Normal AFT Model

To examine the performance of the proposed cafta method for the AFT model with a log‐normal distribution, we generate errors through a standard normal distribution. As shown in Table 2, cafta and oracle show almost identical performances. Compared to the Weibull AFT model results above, the performance of the meta‐estimation for the log‐normal AFT model is even worse, especially for $\beta_{6}$, the coefficient for a categorical variable, with coverage probabilities equal to 68.6%, 81.2%, and 86.6%, which are significantly below the nominal 95% level. Furthermore, the type I error for the likelihood ratio test $\Lambda_{\text{log‐normal}},q=0$ is well controlled at 4.9% and 5.8% for 30% and 50% event rates, respectively. We find that the type I error for the case with 10% event rate shows the unstable behavior of the GG AFT model near q=0 with poor numerical performance in the case of small sample sizes, and thus the results are omitted.

**TABLE 2:** Simulation results from the log‐normal AFT model summarized by all cases of successful convergence over 3000 repetitions. K=6 with the fixed updating order of $n_{1}=500$, $n_{2}=300$, $n_{3}=100$, $n_{4}=50$, and $n_{5}=50$. The true values of $\beta_{1}=0.15$ and $\beta_{6}=0.3$. Refer to the footnotes for the definitions of each metric.

		$\beta_{1}(0.15)$			$\beta_{6}(0.3)$		
Event rate	Metrics	Oracle	CAFTA	Meta	Oracle	CAFTA	Meta
10%	ARB(%)	0.0	3.5	19.1	0.0	11.0	181.9
	CP(%)	94.6	94.1	87.7	93.9	93.5	68.6
	$\text{MSE}\times 10^{-3}$	2.48	2.39	3.29	28.3	26.8	91.2
	$\text{ASE}\times 10^{-2}$	4.91	4.83	4.49	15.9	15.6	14.4
	$\text{ESE}\times 10^{-2}$	4.98	4.87	5.68	16.8	16.3	28.4
	fails(#)	0	0	1891	0	0	1891
30%	ARB(%)	0.0	1.0	7.5	0.0	2.0	43.5
	CP(%)	94.7	94.5	91.6	94.1	94.1	81.2
	$\text{MSE}\times 10^{-3}$	1.27	1.26	1.43	14.5	14.3	26.9
	$\text{ASE}\times 10^{-2}$	3.51	3.50	3.37	11.5	11.4	10.8
	$\text{ESE}\times 10^{-2}$	3.57	3.55	3.77	12.0	12.0	15.8
	fails(#)	0	0	47	0	0	47
50%	ARB(%)	0.0	0.6	5.2	0.0	1.0	21.2
	CP(%)	94.6	94.5	92.8	94.1	94.0	86.6
	$\text{MSE}\times 10^{-4}$	9.48	9.45	10.3	109	109	164
	$\text{ASE}\times 10^{-2}$	3.06	3.05	2.98	10.1	10.0	9.51
	$\text{ESE}\times 10^{-2}$	3.08	3.07	3.21	10.5	10.4	12.7
	fails(#)	0	0	32	0	0	32

*Note:* ARB, average absolute relative bias with respect to the oracle estimate; CP, coverage probability; MSE, mean squared bias; ASE, mean estimated standard error of the estimates; ESE, empirical standard error; fails, number of repetitions when oracle and cafta failed to converge and when at least one site failed to converge for meta.

#### 5.1.3 Results From the Log‐Logistic AFT Model

To examine the performance of the proposed cafta method in the log‐logistic AFT model, errors are generated through a standard logistic distribution. The comparison of the performance shown in Table 3 between cafta, the oracle, and meta estimations is similar to that for the Weibull AFT model and log‐normal AFT model above.

**TABLE 3:** Simulation results for log‐logistic AFT model summarized by all cases of successful convergence over 3000 repetitions. K=6 with the fixed updating order of $n_{1}=500$, $n_{2}=300$, $n_{3}=100$, $n_{4}=50$, and $n_{5}=50$. The true values of $\beta_{1}=0.15$ and $\beta_{6}=0.3$. Refer to the footnotes for the definitions of each metric.

		$\beta_{1}(0.15)$			$\beta_{6}(0.3)$		
Event rate	Metrics	Oracle	CAFTA	Meta	Oracle	CAFTA	Meta
10%	ARB(%)	0.0	13.5	78.9	0.0	39.6	315.4
	CP(%)	95.9	95.9	91.8	95.7	95.4	89.4
	$\text{MSE}\times 10^{-3}$	7.60	7.13	9.08	87.8	79.9	135
	$\text{ASE}\times 10^{-2}$	8.86	8.71	8.38	29.3	28.6	28.9
	$\text{ESE}\times 10^{-2}$	8.72	8.43	9.48	29.6	28.3	36.1
	fails(#)	0	2	1365	0	2	1365
30%	ARB(%)	0.0	2.4	16.0	0.0	9.2	88.9
	CP(%)	95.0	95.0	94.0	95.1	95.3	92.5
	$\text{MSE}\times 10^{-3}$	3.54	3.48	3.70	37.6	36.8	44.3
	$\text{ASE}\times 10^{-2}$	5.92	5.90	5.83	19.4	19.3	19.0
	$\text{ESE}\times 10^{-2}$	5.95	5.90	6.07	19.4	19.2	21.0
	fails(#)	0	0	35	0	0	35
50%	ARB(%)	0.0	1.0	9.5	0.0	16.4	83.7
	CP(%)	95.2	95.2	94.8	95.1	94.9	92.6
	$\text{MSE}\times 10^{-3}$	2.55	2.53	2.71	27.7	27.5	32.7
	$\text{ASE}\times 10^{-2}$	5.06	5.04	5.03	16.7	16.7	16.3
	$\text{ESE}\times 10^{-2}$	3.70	3.70	3.87	12.3	12.3	13.5
	fails(#)	0	0	32	0	0	32

*Note:* ARB, average absolute relative bias with respect to the oracle estimate; CP, coverage probability; MSE, mean squared bias; ASE, mean estimated standard error of the estimates; ESE, empirical standard error; fails, number of repetitions when oracle and cafta failed to converge and when at least one site failed to converge for meta.

## 6 Data Application

We illustrate the proposed cafta method to analyze the Scientific Registry of Transplant Recipients (SRTR) data. The primary goal of the analysis is to assess risk factors associated with the 5‐year death‐censored graft failure (DCGF) for patients who underwent kidney transplants in the US. This analysis begins with a sample size of 62684 patients who received first‐time kidney transplants between 2003 to 2006 from the Organ Procurement and Transplantation Network (OPTN), including K=11 geographic regions across the US. A set of 695 patients who died on the same day of the transplant was removed from the analysis. We use online‐learning based Schoenfeld residuals test proposed by Xue et al. [26] to assess the proportionality assumption of the Cox model and detect relevant evidence for violation of this assumption. This leads to our choice of AFT models to analyze the data.

The overall 5‐year DCGF rate is 13.5% across all 11 regions, with Region 6 having the lowest rate of 10.0% with only 1752 transplant cases, while Region 2 has the highest rate of 16.4% with 6200 transplant cases. We include the following covariates in the AFT model: Age, gender, BMI, race, and ethnicity of donors and recipients, whether the living donor or deceased donor, diabetes and hypertension status of the recipients, and the human leukocyte antigen (HLA) matching type between donors and recipients. The proportion of missingness in the covariates is mild; thus, we conduct a complete‐case analysis including 48766 participants with no missing data. The K=11 OPTN regions are descendingly ordered according to the number of transplant recipients who experience the 5‐year DCGF outcome.

**FIGURE 1:**
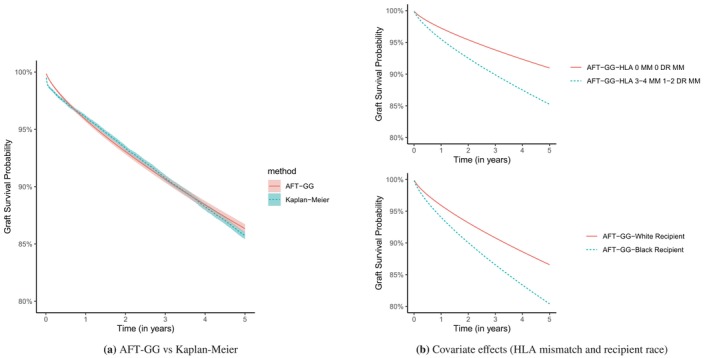
(a) The estimated survival curves obtained by the Kaplan‐Meier method for the pooled data and the fitted survival curves generated from the chosen AFT‐GG model ( $\hat{q}=2.53$ ). (b) Impact of HLA mismatch and recipient race on graft survival probability over time. The top panel shows the effects of HLA mismatch levels on graft survival, comparing recipients with 0 MM 0 DR MM to those with 3‐4 MM 1‐2 DR MM. The bottom panel illustrates the effects of recipient race on graft survival, comparing White and Black recipients.

We apply cafta without requiring subject‐level data sharing for the four candidate AFT models. To make a model choice, we conduct the log‐likelihood test and report AIC: See Table 4 for the results. A GG model of $\hat{q}=2.53$ , with a 95% confidence interval of (2.41, 2.65) is selected with the lowest AIC and is also supported by the likelihood ratio test results of rejecting $H_{0}:q_{0}=1$ (Weibull) and $H_{0}:q_{0}=0$ (log‐normal). We also perform a centralized analysis of the pooled data for the GG model as the gold standard for benchmarking, and both Meta‐FE and Meta‐RE based on OPTN‐region‐specific estimates for comparison in Table 5. Overall, the findings are consistent with the results in obtained using the collaborative Cox proportional hazards model in Hu et al. [5] The estimated coefficients of the oracle and cafta methods are close, while the estimations of the coefficients by the meta method have slightly more deviations from the oracle method with the most dissimilar ones being the coefficients associated with the other category in donor race, 0.93 and 0.92 for the oracle and cafta 0.83 and 0.81 for the two Meta versions. Figure 1a shows a comparison between the empirical Kaplan‐Meier survival curve on the pooled data and the fitted survival curve generated from the chosen AFT‐GG model.

**TABLE 4:** Goodness‐of‐fit results from candidate AFT models for the SRTR data analysis.

AFT models	AIC	LRT ($-\log_{10}(p)$ for $H_{0}:q=q_{0}$)
Weibull $\left(q_{0}=1\right)$	54 141	16
Log‐normal $\left(q_{0}=0\right)$	54 953	193
Log‐logistic	54 246	—
GG ($\hat{q}=2.53$)	54 073	—

**TABLE 5:** Results from a chosen GG AFT model ($\hat{q}=2.53$) for the DCGF outcome from the competing estimation methods.

	Oracle ($\hat{q}=2.55$)		CAFTA ($\hat{q}=2.53$)		Meta‐FE ($\hat{q}=1.73$)		Meta‐RE ($\hat{q}=1.92$)	
	exp(β)	z‐score	exp(β)	z‐score	exp(β)	z‐score	exp(β)	z‐score
Recipient age	1.02	31.57	1.02	22.39	1.03	24.83	1.03	16.97
Donor age	0.98	−26.10	0.98	−16.32	0.98	−17.96	0.98	−12.10
Donor‐recipient gender match (baseline: F‐F)								
F‐M	0.91	−*2.08*	0.91	−*2.21*	0.93	−*1.48*	0.93	−*1.44*
M‐M	1.16	3.09	1.16	3.33	1.16	3.11	1.13	2.02
M‐F	0.98	−0.38	0.99	−0.28	1.00	−0.03	0.97	−0.50
Recipient obesity	0.82	−5.55	0.83	−5.80	0.81	−5.81	0.81	−5.40
Donor obesity	0.94	−1.62	0.94	−1.74	0.96	−1.20	0.96	−0.79
Recipient race (baseline: White)								
Black	0.59	−13.66	0.60	−14.32	0.60	−12.62	0.60	−9.05
Hispanic	1.13	2.08	1.11	1.83	1.00	0.06	1.01	0.08
Other	1.32	3.53	1.31	3.54	1.25	2.66	1.28	2.43
Donor race (baseline: White)								
Black	0.72	−7.46	0.73	−7.37	0.74	−6.55	0.71	−5.67
Hispanic	0.96	−0.77	0.96	−0.81	0.93	−1.26	0.92	−1.22
Other	0.93	−*0.79*	0.92	−*0.81*	0.83	−*1.96*	0.81	−*1.45*
HLA mismatch (baseline: 0 MM 0 DR MM)								
1‐2 MM 0 DR MM	0.75	−2.86	0.75	−2.96	0.75	−2.76	0.76	−2.55
1‐2 MM 1‐2 DR MM	0.58	−7.21	0.59	−7.51	0.59	−6.97	0.59	−5.93
3‐4 MM 0 DR MM	0.67	−4.00	0.67	−4.55	0.64	−4.32	0.68	−3.05
3‐4 MM 1‐2 DR MM	0.51	−9.58	0.52	−9.89	0.51	−9.42	0.51	−8.31
Donor type (deceased)	0.48	−17.86	0.48	−18.01	0.48	−17.15	0.49	−12.44
Recipient diabetes	1.04	1.04	1.04	1.11	1.05	1.18	1.05	0.94
Recipient hypertension	0.94	−1.51	0.94	−1.61	0.95	−1.19	0.94	−0.88

*Note:* Where different significance conclusions across methods are shown by italic values.

The statistical significance conclusions for the oracle and cafta agree on all coefficients except for being a Hispanic donor: The oracle estimate is significant at 95% confidence level, whereas the cafta estimate is significant at 90% confidence level but insignificant at 95% confidence level with a z‐score of 1.83. The Meta‐FE and Meta‐RE methods conclude the effect of being a Hispanic donor to be insignificant, with a z‐score of 0.06 and 0.08, respectively. In addition, both meta methods fail to detect the significant effect of having a female recipient and a male donor. Interestingly, the Meta‐FE method concludes that having a donor of another race (non‐white, non‐black, and non‐Hispanic) is a significant risk factor associated with the DCGF outcome compared to having a white donor. This contradicts the oracle method, the cafta method, and the Meta‐RE method.

The interpretation of the exponentiated AFT coefficients is different from that in terms of hazard ratios. For instance, one additional year of recipient age is significantly associated with a 2% increase in the time to DCGF. However, one additional year of donor age is significantly associated with a 2% decrease in the time to DCGF, making it a risk factor for the DCGF outcome. The other significant risk factors for the DCGF outcome include gender mismatches, obesity, being a Black recipient, having a non‐White donor, HLA mismatches, having a deceased donor, and recipient hypertension. Among them, being a black recipient is associated with a 41% faster progression in pace in terms of time to DCGF compared to a white recipient; having the most HLA mismatch is associated with a 49% faster decaying time to DCGF; having a deceased donor is associated with a 52% faster time to DCGF. The effects of these covariates on graft survival probability are illustrated in Figure 1b.

## 7 Discussion

We apply cafta to the SRTR dataset and present the significant risk factors associated with 5‐year DCGF for patients who underwent kidney transplants. In this project, we present a privacy‐aware collaborative AFT modeling approach without making the PH assumption, termed cafta, that enables a distributed time‐to‐event regression analysis across multiple sites and avoids sharing individual‐level data. We show both theoretically and numerically the desired asymptotic properties of cafta and the proposed likelihood ratio test within the family of parametric generalized gamma (GG) AFT models. The cafta estimation should only proceed on compatible sites unless some extensions are added to the current form of cafta. We consider a family of distributions for the parametric AFT models.

We provide some practical guidance and technical considerations when applying cafta. First, to ensure the numerical stability of cafta, we recommend beginning the cafta procedure with the site that has the largest sample size or the highest number of events, thereby minimizing the risk of numerical issues (e.g., non‐convergence) arising from poor initial values. Second, if the sample sizes and event rates are similar across sites, the cafta estimation results are expected to be stable regardless of the pre‐fixed stream of sites for the sequential updates, evidenced by the numerical study given in Hu et al. [19] To test whether the data collected across sites are homogenous in the model parameters, a key assumption of the cafta framework, we recommend leveraging the model diagnostic procedure in [19] originally designed for checking the compatibility of parameters in the propensity score model. The presence of potential cross‐region heterogeneity highlights the need for caution in interpreting aggregated population‐level results obtained in a population‐average paradigm. Future research could explore causes for heterogeneity, followed by approaches to account for heterogeneity in data analyses. Such an extension can help strengthen the robustness and generalizability of the findings. Alternatively, there are also semi‐parametric AFT models that are technically more demanding, because they involve comparing the residuals across sites. cafta may not be easily extended to these models. More recently, Pang et al. [27] and Crowther et al. [28] have proposed flexible AFT models that approximate the baseline hazards using polynomial splines. The added flexibility from polynomial splines allows the model to accommodate more complex hazard shapes and may potentially improve the model fit to the data. Future works may add such a flexible AFT model into cafta. Additionally, graphical diagnosis can also be used for goodness‐of‐fit evaluation, which is popular among practitioners. One way is a graphical comparison between the empirical Kaplan‐Meier survival curve and the fitted survival curve generated from a chosen AFT model. There has been some recent interest in constructing a private empirical survival curve [29, 30, 31]. Future work may investigate the utility of these procedures in constructing collaborative diagnostics plots in the cafta paradigm. To facilitate the implementation of cafta, we develop an R package CAFTA available at the website (https://github.com/CollaborativeInference) to streamline data analysis and summary statistics sharing under the collaborative inference framework. Within cafta, there remains room for improving communication efficiency. Hector et al. [32] proposed a Parallel‐and‐Stream Accelerator (PASA) paradigm that leverages the strength of both distributed strategy and sequential strategy for computationally fast, statistically efficient, and scalable learning. For example, in geographically clustered regions with established close collaboration, sequential processing can be used locally for cafta, while distributed computing can be employed across regions to reduce communication overhead.

## Conflicts of Interest

The authors declare no conflicts of interest.

## Supporting information




**Data S1.** Supporting Information.

## Data Availability

The R package for simulating and performing cafta is available online at https://github.com/CollaborativeInference/CAFTA. Patient‐level real‐world data can be obtained by submitting a request to the Scientific Registry of Transplant Recipients (SRTR).
