# Supplementary material for: Collaborative Inference for Accelerated Failure Time Model Using Clinical Center‐Level Summary Statistics
Source: Stat Med. 2025 Oct 22;44(23-24):e70279. doi: 10.1002/sim.70279 (PMC12542814; doi:10.1002/sim.70279)
Supplement: Supplementary file 1 — Data S1. Supporting Information. [file SIM-44-0-s001.pdf]

Collaborative Inference for Accelerated Failure Time Model Using Clinical Center-level Summary Statistics

Mengtong Hu, Xu Shi, Ziyang Gong, and Peter X.-K. Song
Department of Biostatistics, University of Michigan

September, 2025

Web Appendix A Derivations for Weibull, log-logistic, and log-normal AFT models

Weibull AFT models. With a slight abuse of notation, we present the results for an arbitrary individual i. We consider the vector X_i to include 1, so that α is part of β. The likelihood function is

$$l_i(\theta) = \Delta_i\{W_i - \log(\sigma)\} - e^{W_i}.$$

The two components of the score function are

$$\frac{\partial l(\theta)}{\partial \beta} = \sigma^{-1} X_i (e^{W_i} - \Delta_i),$$
$$\frac{\partial l(\theta)}{\partial \sigma} = \sigma^{-1}\{W_i(e^{W_i} - \Delta_i) - \Delta_i\}.$$

The entries in the sensitivity matrix are

$$-\frac{\partial^2 l(\theta)}{\partial \beta \partial \beta^{\mathrm{T}}} = \sigma^{-2} X_i X_i^T e^{W_i},$$
$$-\frac{\partial^2 l(\theta)}{\partial \beta \partial \sigma} = \sigma^{-2} X_i W_i e^{W_i} + \sigma^{-2} X_i (e^{W_i} - \Delta_i) = \sigma^{-2} X_i (W_i e^{W_i} + e^{W_i} - \Delta_i),$$
$$-\frac{\partial^2 l(\theta)}{\partial \sigma \partial \sigma} = \sigma^{-2}(W_i^2 e^{W_i} - \Delta_i) + 2\sigma^{-2} W_i (e^{W_i} - \Delta_i).$$

Log-logistic AFT models. The likelihood function is

$$l_i(\theta) = \Delta_i\{W_i - \log(1 + e^{W_i}) - \log(\sigma)\} - \log(1 + e^{W_i})$$

The two components of the score function are

$$\frac{\partial l(\theta)}{\partial \beta} = \sigma^{-1} X_i(-\Delta_i + (1+\Delta_i)e^{W_i}(1+e^{W_i})^{-1}),$$
$$\frac{\partial l(\theta)}{\partial \sigma} = \sigma^{-1}[W_i\{-\Delta_i + (1+\Delta_i)e^{W_i}(1+e^{W_i})^{-1}\} - \Delta_i].$$

The entries in the sensitivity matrix H are

$$-\frac{\partial^2 l(\theta)}{\partial \beta \partial \beta^{\mathrm{T}}} = \sigma^{-2} X_i X_i^T (1+\Delta_i)e^{W_i}(1+e^{W_i})^{-2},$$
$$-\frac{\partial^2 l(\theta)}{\partial \beta \partial \sigma} = \sigma^{-2} X_i W_i (1+\Delta_i)e^{W_i}(1+e^{W_i})^{-2} + \sigma^{-2} X_i(-\Delta_i + (1+\Delta_i)e^{W_i}(1+e^{W_i})^{-1}),$$
$$-\frac{\partial^2 l(\theta)}{\partial \sigma \partial \sigma} = \sigma^{-2}(W_i^2(1+\Delta_i)e^{W_i}(1+e^{W_i})^{-2} - \Delta_i) + 2\sigma^{-2} W_i(-\Delta_i + (1+\Delta_i)e^{W_i}(1+e^{W_i})^{-1}).$$

Log-normal AFT models. Denote the density function of the standard normal distribution by $\phi(z)$ and the cumulative density function of the standard normal distribution by $\Phi(z)$. The likelihood function is

$$l_i(\theta) = \Delta_i(\log(\phi(W_i)) - \log(\sigma)) + (1-\Delta_i)(\log(1-\Phi(W_i))).$$

The two components of the score function are

$$\frac{\partial l(\theta)}{\partial \beta} = \sigma^{-1} X_i(\Delta_i W_i + (1-\Delta_i)\phi(W_i)\Phi^{-1}(W_i)),$$
$$\frac{\partial l(\theta)}{\partial \sigma} = \sigma^{-1}\{W_i^2 \Delta_i + W_i(1-\Delta_i)\phi(W_i)\Phi^{-1}(W_i) - \Delta_i\}.$$

The entries in the sensitivity matrix H are

$$-\frac{\partial^2 l(\theta)}{\partial \beta \partial \beta^{\mathrm{T}}} = \sigma^{-2} X_i X_i^T (\Delta_i + (1-\Delta_i)\phi(W_i)\Phi^{-1}(W_i)[\phi(W_i)\Phi^{-1}(W_i) - W_i]),$$
$$-\frac{\partial^2 l(\theta)}{\partial \beta \partial \sigma} = 2\sigma^{-2} X_i W_i \Delta_i + \sigma^{-2} X_i (1-\Delta_i)\phi(W_i)\Phi^{-1}(W_i)[\{W_i\phi(W_i)\Phi^{-1}(W_i) - W_i^2\} + 1],$$
$$-\frac{\partial^2 l(\theta)}{\partial \sigma \partial \sigma} = 3\sigma^{-2} W_i^2 - \sigma^{-2}\Delta_i + \sigma^{-2} W_i(1-\Delta_i)\phi(W_i)\Phi^{-1}(W_i)\{W_i\phi(W_i)\Phi^{-1}(W_i) - W_i^2 + 2\}.$$

The variability matrix for the three models above can be calculated using the outer product of the score functions, respectively.

Web Appendix B Proofs of large-sample properties

Proof of Theorem 1. Theorem 1 is proved by the method of induction. Assume that the previous $\hat{\theta}_j$ are consistent for θ_0, for $j = 1, \ldots, k-1$. Let the current update be $\hat{\theta}_k$ as a solution to equation (6). Rewriting equation (6) with θ_0 according to the Law of Large Numbers, by Assumption 1 we

obtain

$$\frac{1}{N_k}U_k(\theta_0) + \frac{1}{N_k}\sum_{j=1}^{k-1} H_j(\hat{\theta}_j)(\hat{\theta}_{k-1} - \theta_0) = o_p(1). \qquad \text{(Web Appendix B1)}$$

Taking the first-order multi-dimensional Taylor series on the term $U_k(\hat{\theta}_k)$ around θ_0 leads to, under 1(b),

$$U_k(\hat{\theta}_k) - U_k(\theta_0) = -H_k(\xi_k)(\hat{\theta}_k - \theta_0) = -\{H_k(\theta_0) - H_k(\theta_0) + H_k(\xi_k)\}(\hat{\theta}_k - \theta_0), \qquad \text{(Web Appendix B2)}$$

where $\xi = t\hat{\theta}_k + (1-t)\theta_0$ for some $t \in (0, 1)$. It follows from the Lipschitz continuity (Condition 1(c)) that there exists a constant M_{n_k} such that

$$\|H_k(\xi_k) - H_k(\theta_0)\| \le M_{n_k}\|\xi_k - \theta_0\| \le M_{n_k}\|\hat{\theta}_k - \theta_0\|,$$

where M_{n_k} is bounded by the local sample size n_k. Then equation (Web Appendix B2) can be re-expressed as follows

$$U_k(\hat{\theta}_k) - U_k(\theta_0) = -H_k(\theta_0)(\hat{\theta}_k - \theta_0) + O_p(n_k\|\hat{\theta}_k - \theta_0\|^2). \qquad \text{(Web Appendix B3)}$$

Multiply equation (4) by $1/N_k$ and take the difference from (Web Appendix B1), we have

$$\frac{1}{N_k}\left\{U_k(\theta_0) - U_k(\hat{\theta}_k)\right\} + \frac{1}{N_k}\sum_{j=1}^{k-1} H_j(\hat{\theta}_j)(\hat{\theta}_k - \theta_0) = o_p(1). \qquad \text{(Web Appendix B4)}$$

According to equation (Web Appendix B3), equation (Web Appendix B4) becomes

$$\frac{1}{N_k}\left\{\sum_{j=1}^{k-1} H_j(\hat{\theta}_j) + H_k(\theta_0)\right\}(\hat{\theta}_k - \theta_0) + O_p(\frac{n_k}{N_k}\|\hat{\theta}_k - \theta_0\|^2) = o_p(1). \qquad \text{(Web Appendix B5)}$$

This implies that the leading term must be $o_p(1)$. By Assumption 1 and the assumption that $\hat{\theta}_j$ is consistent for $j = 1, \cdots, k-1$, $\hat{\theta}_k \xrightarrow{p} \theta_0$. □

Proof of Theorem 2. Theorem 2 establishes the asymptotic normality of $\hat{\theta}_k$. First we look at the estimating function (4) for site k. Plugging equation (Web Appendix B3) leads to

$$\begin{aligned} U_k(\theta_0) &= -\sum_{j=1}^{k-1} H_j(\hat{\theta}_j)(\hat{\theta}_{k-1} - \hat{\theta}_k) + H_k(\theta_0)(\hat{\theta}_k - \theta_0) + O_p(n_k\|\hat{\theta}_k - \theta_0\|^2) \\ &= \left\{\sum_{j=1}^{k-1} H_j(\hat{\theta}_j) + H_k(\theta_0)\right\}(\hat{\theta}_k - \theta_0) - \sum_{j=1}^{k-1} H_j(\hat{\theta}_j)(\hat{\theta}_{k-1} - \theta_0) + O_p(n_k\|\hat{\theta}_k - \theta_0\|^2). \end{aligned} \qquad \text{(Web Appendix B6)}$$

Next we investigate the cumulative estimating function up to site k. We begin with the first two

datasets. Applying equation (Web Appendix B6) for the first two datasets, consequently, we have

$$U_1(\theta_0) = H_1(\hat{\theta}_1)(\hat{\theta}_1 - \theta_0) + O_p(n_1\|\hat{\theta}_1 - \theta_0\|^2), \qquad \text{(Web Appendix B7)}$$

and

$$U_2(\theta_0) = \left\{H_1(\hat{\theta}_1) + H_2(\theta_0)\right\}(\hat{\theta}_2 - \theta_0) - H_1(\hat{\theta}_1)(\hat{\theta}_1 - \theta_0) + O_p(n_2\|\hat{\theta}_2 - \theta_0\|^2). \qquad \text{(Web Appendix B8)}$$

Summing up equation (Web Appendix B7) and (Web Appendix B8) leads to

$$\sum_{j=1}^{2} U_j(\theta_0) = \left\{H_1(\hat{\theta}_1) + H_2(\theta_0)\right\}(\hat{\theta}_2 - \theta_0) + O_p(\sum_{j=1}^{2} n_j\|\hat{\theta}_j - \theta_0\|^2).$$

Repeating the same procedure up to the kth dataset, we obtain

$$\sum_{j=1}^{k} U_j(\theta_0) = \left\{\sum_{j=1}^{k-1} H_j(\hat{\theta}_j) + H_k(\theta_0)\right\}(\hat{\theta}_k - \theta_0) + O_p\left(\sum_{j=1}^{k} n_j\|\hat{\theta}_j - \theta_0\|^2\right). \qquad \text{(Web Appendix B9)}$$

Under the Lipschitz continuity condition 1(c), we can substitute $\sum_{j=1}^{k-1} H_j(\hat{\theta}_j)$ by $\sum_{j=1}^{k-1} H_j(\theta_0)$ with the order of error $O_p(\sum_{j=1}^{k} n_j\|\hat{\theta}_j - \theta_0\|^2)$. Consequently, we have

$$\frac{1}{N_k}\sum_{j=1}^{k} U_j(\theta_0) = \left\{\frac{1}{N_k}\sum_{j=1}^{k} H_j(\theta_0)\right\}(\hat{\theta}_k - \theta_0) + O_p\left(\sum_{j=1}^{k} \frac{n_j}{N_k}\|\hat{\theta}_j - \theta_0\|^2\right).$$

Given that $\hat{\theta}_j$ are consistent for θ_0, for $j = 1, \ldots, k-1$, we can see that $1/N_k \sum_{j=1}^{k} U_j(\theta_0)$ and $1/N_k\{\sum_{j=1}^{k} H_j(\theta_0)\}(\hat{\theta}_k - \theta_0)$ are asymptotically equivalent with the same asymptotic distribution. By the Central Limit Theorem, $1/\sqrt{N_k} \sum_{j=1}^{k} U_j(\theta_0) \xrightarrow{d} N(0, V(\theta_0))$, where the variability matrix $V(\theta_0) = E_{\theta_0}\{U(\theta_0)U^{\mathrm{T}}(\theta_0)\}$. It follows immediately that $1/N_k\{\sum_{j=1}^{k} H_j(\theta_0)\}(\hat{\theta}_k - \theta_0)$ is asymptotically normally distributed with mean 0 and variance $V(\theta_0)$; that is,

$$\frac{1}{N_k}\left\{\sum_{j=1}^{k} H_j(\theta_0)\right\}(\hat{\theta}_k - \theta_0) \xrightarrow{d} N(0, V(\theta_0)), \text{ as } N_k \to \infty.$$

Note that the Weak Law of Large Number, $1/N_k \sum_{j=1}^{k} H_j(\theta_0) \xrightarrow{p} H(\theta_0)$. Moreover, we obtain $\sqrt{N_k}(\hat{\theta}_k - \theta_0) \xrightarrow{d} N(0, J(\theta_0))$, where $J(\theta_0) = H(\theta_0)^{-1}V(\theta_0)\{H(\theta_0)^{-1}\}^{\mathrm{T}}$. □

Proof of Theorem 3. Theorem 3 establishes the asymptotic equivalence. We compare the convergence rate of incremental estimator $\hat{\theta}_k$ and the oracle estimator $\hat{\theta}^{ora}$ computed on the pooled data set. The estimating equation for $\hat{\theta}^{ora}$ is $\sum_{j=1}^{k} U_j(\hat{\theta}^{ora}) = 0$. Taking a Taylor expansion around θ_0, we have

$$\sum_{j=1}^{k} U_j(\hat{\theta}^{ora}) = \sum_{j=1}^{k} U_j(\theta_0) - \sum_{j=1}^{k} H_{n_j}(\theta_0)(\hat{\theta}^{ora} - \theta_0) + O_p(N_k \|\hat{\theta}^{ora} - \theta_0\|^2) = 0.$$

(Web Appendix B10)

Taking the difference of equation (Web Appendix B9) and (Web Appendix B10), we have:

$$\frac{1}{N_k} \sum_{j=1}^{k} H_j(\theta_0)(\hat{\theta}_k - \hat{\theta}^{ora}) = O_p(\sum_{j=1}^{k} \frac{n_j}{N_k} \|\hat{\theta}_j - \theta_0\|^2 + \|\hat{\theta}^{ora} - \theta_0\|^2).$$

The Central Limit Theorem gives $\|\hat{\theta}^{ora} - \theta_0\| = O_p(1/\sqrt{N_k})$ and the asymptotic normality in Theorem 2 also gives $\|\hat{\theta}_j - \theta_0\| = O_p(1/\sqrt{N_j})$ for $j = 1, \ldots, k$. Thus, $\|\hat{\theta}_k - \hat{\theta}^{ora}\| = O_p(1/N_k \sum_{j=1}^{k} n_j/N_j)$. From the fact that $\sum_{j=1}^{k} n_j/N_j \leq 1 + log(N_k/N_1)$, $\|\hat{\theta}_k - \hat{\theta}^{ora}\|$ yields the order of $O_p(log(N_k)/N_k)$; that is,

$$\|\hat{\theta}_k - \hat{\theta}^{ora}\|^2 = O_p(2log(N_k)/N_K^2) = O_p(1/N_k)o_p(1) = o_p(1/N_k).$$

□

Proof of Theorem 4. We prove the asymptotic distribution for $\Lambda_{k,\mathrm{GG}(q_0)}$. The log-likelihood ratio test statistics is estimated as

$$\Lambda_{k,\mathrm{GG}(q_0)} = -2\left\{\ell(\hat{\theta}_{k,\mathrm{GG}(q_0)}) - \ell(\hat{\theta}_{k,GG})\right\}.$$

Adding and subtracting the oracle estimate, we have

$$\Lambda_{k,\mathrm{GG}(q_0)} = -2\left\{\ell(\hat{\theta}_{k,\mathrm{GG}(q_0)}) - \ell(\hat{\theta}^{\mathrm{ora}}_{k,\mathrm{GG}(q_0)}) + \ell(\hat{\theta}^{\mathrm{ora}}_{k,\mathrm{GG}(q_0)}) - \ell(\hat{\theta}^{\mathrm{ora}}_{k,GG}) + \ell(\hat{\theta}^{\mathrm{ora}}_{k,GG}) - \ell(\hat{\theta}_{k,GG})\right\}.$$

By the consistency of $\hat{\theta}_{k,\mathrm{GG}(q_0)}$ and $\hat{\theta}_{k,GG}$ shown in Theorem 1, $\Lambda_{k,\mathrm{GG}(q_0)}$ can be written as

$$\Lambda_{k,\mathrm{GG}(q_0)} = -2\left\{\ell(\hat{\theta}^{\mathrm{ora}}_{k,\mathrm{GG}(q_0)}) - \ell(\hat{\theta}^{\mathrm{ora}}_{k,GG})\right\} + o_p(1).$$

The term on the left-hand side of the plus sign converges in distribution to χ^2 with 1 degree of freedom, and by Slutsky's Theorem we have

$$\Lambda_{k,\mathrm{GG}(q_0)} \xrightarrow{d} \chi^2_1.$$

□

Web Table 1: Addition results for Table 1 from the Weibull AFT model.

		β_2			β_3			β_4			β_5		
Event Rate	*Metrics*	*Oracle*	CAFTA	*Meta*	*Oracle*	CAFTA	*Meta*	*Oracle*	CAFTA	*Meta*	*Oracle*	CAFTA	*Meta*
10%	ARB(%)	0.0	11.4	62.6	0.0	15.4	82.1	0.0	45.8	410.8	0.0	48.4	229.8
	CP(%)	95.4	94.5	91.0	94.9	95.2	92.6	96.1	95.6	90.1	95.6	95.0	89.9
	MSE $\times 10^{-3}$	6.75	6.38	24.7	33.6	31.3	3720	91.7	82.9	2802	70.1	63.0	1086
	ASE $\times 10^{-2}$	8.26	8.06	7.99	18.2	17.8	18.3	29.8	29.1	30.2	26.0	25.3	26.3
	ESE $\times 10^{-2}$	8.22	7.97	15.7	18.3	17.7	193	30.3	28.8	167	26.4	25.1	104
30%	ARB(%)	0.0	2.0	11.1	0.0	16.2	60.1	0.0	129.0	654.6	0.0	211.3	738.2
	CP(%)	94.5	94.5	92.8	95.2	95.2	92.9	95.0	94.8	92.7	95.2	95.0	90.6
	MSE $\times 10^{-3}$	2.39	2.34	2.64	11.3	11.1	13.0	30.8	29.9	35.6	23.7	23.0	29.6
	ASE $\times 10^{-2}$	4.80	4.77	4.75	10.7	10.6	10.6	17.1	17.0	17.0	15.1	15.0	14.8
	ESE $\times 10^{-2}$	4.89	4.84	5.13	10.6	10.6	11.3	17.5	17.3	18.9	15.4	15.2	16.9
50%	ARB(%)	0.0	0.9	7.1	0.0	1.1	10.4	0.0	31.4	279.6	0.0	45.4	214.3
	CP(%)	94.1	94.2	92.6	95.1	95.0	92.2	94.6	94.7	91.6	94.6	94.3	89.9
	MSE $\times 10^{-3}$	1.42	1.41	1.56	7.07	7.03	8.45	18.0	17.8	21.3	14.5	14.4	18.8
	ASE $\times 10^{-2}$	3.69	3.68	3.65	8.39	8.34	8.26	13.2	13.2	12.9	11.8	11.7	11.4
	ESE $\times 10^{-2}$	3.77	3.76	3.95	8.41	8.38	9.06	13.4	13.4	14.6	12.0	12.0	13.4

ARB, average absolute relative bias with respect to the oracle estimate; CP, coverage probability; MSE, mean squared bias; ASE, mean estimated standard error of the estimates; ESE, empirical standard error; FAILS, number of repetitions when oracle and CAFTA failed to converge and when at least one site failed to converge for meta.

Web Table 2: Addition results for Table 1 from the log-normal AFT model.

		β_2			β_3			β_4			β_5		
Event Rate	*Metrics*	*Oracle*	CAFTA	*Meta*	*Oracle*	CAFTA	*Meta*	*Oracle*	CAFTA	*Meta*	*Oracle*	CAFTA	*Meta*
10%	ARB(%)	0.0	3.5	19.6	0.0	3.9	33.4	0.0	36.6	450.4	0.0	11.0	181.9
	CP(%)	94.3	94.3	86.0	94.7	94.5	85.0	94.2	93.8	72.4	93.9	93.5	68.6
	MSE $\times 10^{-3}$	2.57	2.46	3.54	12.1	11.6	19.3	25.7	24.2	74.9	28.3	26.8	91.2
	ASE $\times 10^{-2}$	4.92	4.84	4.48	10.8	10.6	10.0	15.2	14.9	13.9	15.9	15.6	14.4
	ESE $\times 10^{-2}$	5.07	4.94	5.88	11.0	10.7	13.7	16.0	15.5	25.9	16.8	16.3	28.4
30%	ARB(%)	0.0	1.0	7.1	0.0	1.1	10.2	0.0	19.9	212.0	0.0	2.0	43.5
	CP(%)	94.6	94.7	91.9	95.1	95.1	91.7	94.1	94.1	82.7	94.1	94.1	81.2
	MSE $\times 10^{-3}$	1.27	1.25	1.41	6.53	6.49	7.78	13.9	13.7	25.0	14.5	14.3	26.9
	ASE $\times 10^{-2}$	3.52	3.50	3.38	8.03	7.99	7.70	11.3	11.3	10.6	11.5	11.4	10.8
	ESE $\times 10^{-2}$	3.56	3.54	3.75	8.08	8.05	8.81	11.8	11.7	15.5	12.0	12.0	15.8
50%	ARB(%)	0.0	0.5	5.1	0.0	0.6	7.2	0.0	8.7	200.1	0.0	1.0	21.2
	CP(%)	95.2	95.3	92.9	95.3	95.1	92.7	94.0	94.0	87.2	94.1	94.0	86.6
	MSE $\times 10^{-3}$	0.95	0.94	1.03	5.06	5.05	5.77	10.7	10.7	16.2	10.9	10.9	16.4
	ASE $\times 10^{-2}$	3.07	3.05	2.99	7.16	7.13	6.95	10.1	10.0	9.5	10.1	10.0	9.51
	ESE $\times 10^{-2}$	3.08	3.07	3.21	7.12	7.11	7.60	10.3	10.3	12.7	10.5	10.4	12.7

ARB, average absolute relative bias with respect to the oracle estimate; CP, coverage probability; MSE, mean squared bias; ASE, mean estimated standard error of the estimates; ESE, empirical standard error; FAILS, number of repetitions when oracle and CAFTA failed to converge and when at least one site failed to converge for meta.

Web Table 3: Addition results for Table 3 from the Log-logistic AFT model.

		β_2			β_3			β_4			β_5		
Event Rate	*Metrics*	*Oracle*	CAFTA	*Meta*	*Oracle*	CAFTA	*Meta*	*Oracle*	CAFTA	*Meta*	*Oracle*	CAFTA	*Meta*
10%	ARB(%)	0.0	11.3	65.7	0.0	86.0	1569.2	0.0	76.2	505.0	0.0	49.0	255.8
	CP(%)	94.2	94.2	90.4	94.8	95.1	92.4	95.9	95.7	89.7	95.6	95.5	89.0
	MSE $\times 10^{-3}$	8.56	8.03	10.1	40.3	37.5	48.0	103	94.1	166	82.1	74.2	125
	ASE $\times 10^{-2}$	8.88	8.72	8.41	19.9	19.6	19.6	32.1	31.5	31.9	28.4	27.8	28.1
	ESE $\times 10^{-2}$	9.25	8.94	9.97	20.1	19.3	21.9	32.1	30.7	40.5	28.6	27.2	35.4
30%	ARB(%)	0.0	2.4	14.8	0.0	3.4	29.0	0.0	36.3	275.8	0.0	34.1	424.6
	CP(%)	94.9	95.1	94.2	94.5	94.5	93.5	95.2	95.5	92.4	95.6	95.4	92.1
	MSE $\times 10^{-3}$	3.53	3.47	3.75	19.4	19.0	20.7	45.7	44.7	55.7	37.1	36.3	45.2
	ASE $\times 10^{-2}$	5.93	5.90	5.85	13.7	13.6	13.5	21.5	21.4	21.4	19.3	19.2	18.9
	ESE $\times 10^{-2}$	5.95	5.89	6.11	13.9	13.8	14.4	21.4	21.1	23.6	19.3	19.0	21.2
50%	ARB(%)	0.0	1.0	9.2	0.0	1.7	17.5	0.0	78.6	402.3	0.0	40.4	396.9
	CP(%)	94.8	94.6	93.9	95.3	95.3	94.3	95.1	95.0	92.7	95.1	95.2	92.0
	MSE $\times 10^{-3}$	2.61	2.59	2.78	14.6	14.6	15.4	34.5	34.2	39.9	28.5	28.4	33.2
	ASE $\times 10^{-2}$	5.07	5.05	5.04	12.0	11.9	11.9	18.6	18.6	18.3	16.9	16.8	16.4
	ESE $\times 10^{-2}$	5.11	5.09	5.27	12.1	12.1	12.4	18.6	18.5	20.0	16.9	16.8	18.2

ARB, average absolute relative bias with respect to the oracle estimate; CP, coverage probability; MSE, mean squared bias; ASE, mean estimated standard error of the estimates; ESE, empirical standard error; FAILS, number of repetitions when oracle and CAFTA failed to converge and when at least one site failed to converge for meta.

Web Appendix C   Additional simulation results

Web Appendix C.1   Results for $\beta_3 \to \beta_5$ for Table 1 to Table3

Web Appendix C.2   Results under varying event rates across sites

We perform simulations in which each site is assigned a distinct censoring rate, resulting in site-specific event rates of 10%, 10%, 30%, 30%, 50%, and 50%. All other settings remain identical to those in the main simulation study. As shown in Table 4, CAFTA consistently achieves coverage close to the nominal level in all the error distributions considered. In contrast, the fixed-effects meta-analysis method (Meta-FE) exhibits increased bias, leading to undercoverage, while the random-effects meta-analysis (Meta-RE) yields inflated variance estimates, resulting in reduced efficiency and potential power loss.

Web Appendix C.3   Results under covariate distribution shifts

We conduct simulations to assess the robustness of our method under covariate distribution shifts across sites. Although the full dataset was generated from the same underlying distribution, we induce dependence of data collection between site assignments and covariates to create site-specific heterogeneity.

To implement this, we first randomly selected 500 individuals from the grand whole dataset to form Site 1. For the remaining individuals, we computed a score for each subject i as a fixed linear

Web Table 4: Simulation results with heterogeneous event rates across all error distributions summarized by all cases of successful convergence over 3000 repetitions. $K = 6$ with the fixed updating order of $n_1 = 500$, $n_2 = 300$, $n_3 = 100$, $n_4 = 50$, and $n_5 = 50$. The true values of $\beta_1 = 0.15$ and $\beta_6 = 0.3$. Refer to the footnotes for the definitions of each metric.

		β_1 (0.15)				β_6 (0.3)			
Distribution	*Metrics*	*Oracle*	CAFTA	*Meta-FE*	*Meta-RE*	*Oracle*	CAFTA	*Meta-FE*	*Meta-RE*
Weibull	ARB(%)	0.0	35.7	306	371	0.0	37.9	147	1090
	CP(%)	95.1	94.3	92.9	97.2	94.4	93.8	89.1	97.7
	MSE $\times 10^{-3}$	4.08	4.09	56.8	5.22	45.3	43.9	292	1610
	ASE $\times 10^{-2}$	6.36	6.17	6.23	8.47	20.8	20.4	20	77.5
	ESE $\times 10^{-2}$	6.38	6.39	23.8	7.23	21.3	20.9	54	115
	FAILS#	1	3	85	87	1	3	85	87
Log-Normal	ARB(%)	0.0	15.3	40.9	64.9	0.0	35.4	123	945
	CP(%)	95.2	94.7	93.4	97.2	94.5	94.6	90	97.7
	MSE $\times 10^{-3}$	5.65	5.56	6.09	7.35	62.5	59.7	82.3	1980
	ASE $\times 10^{-2}$	7.52	7.39	7.36	10	24.8	24.6	24.1	89.8
	ESE $\times 10^{-2}$	7.52	7.46	7.79	8.57	25	24.4	28.7	125
	FAILS#	0	3	45	45	0	3	45	45
Log-Logistic	ARB(%)	0.0	2.96	12.9	17.8	0.0	65	407	834
	CP(%)	94.8	94.2	91.2	96.5	94	93.8	80.8	97.1
	MSE $\times 10^{-3}$	1.92	1.89	2.19	2.78	21.6	21	43.4	173
	ASE $\times 10^{-2}$	4.31	4.23	4.05	5.86	14	13.8	12.8	31.6
	ESE $\times 10^{-2}$	4.38	4.34	4.67	5.27	14.7	14.5	20.3	38
	FAILS#	0	0	36	36	0	0	36	36

ARB, average absolute relative bias with respect to the oracle estimate; CP, coverage probability; MSE, mean squared bias; ASE, mean estimated standard error of the estimates; ESE, empirical standard error; FAILS, number of repetitions when oracle and CAFTA failed to converge and when at least one site failed to converge for meta.

combination of covariates with added Gaussian noise according to the following rule:

$$S_i = \mathbf{x}_i^\top \mathbf{w} + \varepsilon_i, \quad \text{where } \mathbf{w} = (1, -1, 1, -1, -0.5, 0.5)^\top,\ \varepsilon_i \sim \mathcal{N}(0, 1),$$

where $\mathbf{x}_i$ denotes the covariate vector for subject i. Individuals are then sorted by their scores S_i and sequentially assigned to Sites 2 through 6 in equal-sized blocks. This procedure induces systematic variation in covariate distributions across sites, thereby violating the i.i.d. assumption, especially "identically distributed" at locat sites.

Table 5 presents the simulation results. As shown, CAFTA maintains coverage close to the nominal level, even under the covariate shifts. In contrast, both meta-analysis approaches perform less reliably, exhibiting inappropriate coverage and higher estimation errors across all settings.

Web Table 5: Simulation results with covariates shift across all error distributions summarized by all cases of successful convergence over 3000 repetitions. $K = 6$ with the fixed updating order of $n_1 = 500$, $n_2 = 300$, $n_3 = 100$, $n_4 = 50$, and $n_5 = 50$. The true values of $\beta_1 = 0.15$ and $\beta_6 = 0.3$. Refer to the footnotes for the definitions of each metric.

		β_1 (0.15)				β_6 (0.3)			
Distribution	*Metrics*	*Oracle*	CAFTA	*Meta-FE*	*Meta-RE*	*Oracle*	CAFTA	*Meta-FE*	*Meta-RE*
Weibull	ARB(%)	0.0	0.92	11.4	34.4	0.0	3.11	41.2	528
	CP(%)	94.9	94.9	93.4	96.8	94.2	94.1	87.9	97.3
	MSE $\times 10^{-3}$	1.37	1.37	1.75	5.31	15.2	15.1	22.5	1320
	ASE $\times 10^{-2}$	3.69	3.67	4	7.66	12	11.9	11.6	65.4
	ESE $\times 10^{-2}$	3.7	3.7	4.18	7.28	12.3	12.3	14.6	106
	FAILS#	0	0	651	651	0	0	651	651
Log-Normal	ARB(%)	0.0	1.02	18.2	50.4	0.0	15.6	256	5870
	CP(%)	95.2	95.1	94.5	97	95.1	95	89.8	98.2
	MSE $\times 10^{-3}$	2.55	2.54	3.26	8.71	27.7	27.5	41.2	1410
	ASE $\times 10^{-2}$	5.06	5.04	5.53	9.95	16.7	16.7	16.2	70.8
	ESE $\times 10^{-2}$	5.05	5.04	5.71	9.33	16.6	16.6	20.2	109
	FAILS#	0	0	566	566	0	0	566	566
Log-Logistic	ARB(%)	0.0	0.56	9.26	28	0.0	1.05	25.9	105
	CP(%)	94.6	94.5	94.1	96.8	94.1	94	83.8	97.2
	MSE $\times 10^{-3}$	0.95	0.94	1.2	3.57	10.9	10.9	18.8	167
	ASE $\times 10^{-2}$	3.06	3.05	3.29	6.4	10.1	10	9.45	29.3
	ESE $\times 10^{-2}$	3.08	3.07	3.46	5.98	10.5	10.4	13.5	37.4
	FAILS#	0	0	558	558	0	0	558	558

ARB, average absolute relative bias with respect to the oracle estimate; CP, coverage probability; MSE, mean squared bias; ASE, mean estimated standard error of the estimates; ESE, empirical standard error; FAILS, number of repetitions when oracle and CAFTA failed to converge and when at least one site failed to converge for meta.
